# The Early Diagnosis of Scrub Typhus by Metagenomic Next-Generation Sequencing

**DOI:** 10.3389/fpubh.2021.755228

**Published:** 2021-11-11

**Authors:** Xianghong Liu, Ye Zhang, Jun Zhang, Zheng Lou, Han Xia, Zhijuan Lu

**Affiliations:** ^1^Department of Neurology, Ganzhou People's Hospital, Ganzhou, China; ^2^Department of Scientific Affairs, Hugobiotech Co., Ltd., Beijing, China; ^3^Emergency Department, Ganzhou People's Hospital, Ganzhou, China

**Keywords:** scrub typhus, *Orientia tsutsugamushi*, early diagnosis, metagenomic next-generation sequencing, eschars

## Abstract

**Introduction:** Scrub typhus is a mite-borne infection widespread in Southeast Asia, with clinical symptoms such as fever, chills, skin rash, eschar at the bite site, and other signs of acute febrile illness. The *Rickettsia* pathogen (*Orientia tsutsugamushi*) is always difficult to be diagnosed at an early stage by traditional clinical diagnostic methods, especially for patients without typical eschar. This greatly increases the mortality of patients with scrub typhus. A new approach should be introduced to improve its clinical diagnosis.

**Methods:** During May 2018 to March 2021, 13 samples from 10 patients with suspected scrub typhus were collected. Metagenomic next-generation sequencing (mNGS) and other diagnostic methods (including serology using Weil–Felix reaction and indirect immunofluorescence test (IIFT) for scrub typhus and respiratory tract profile IgM as well as culture for routine bacteria) were used to identify the pathogens in this study.

**Results:** The results of mNGS were all positive, with mapped reads of *O. tsutsugamushi* ranging from 1 to 460. Eight patients (80%) were diagnosed as scrub typhus. The other two were diagnosed as suspected scrub typhus due to the limited number of reads of the pathogen (one and two, respectively). According to clinical evidences, nine of the 10 patients were finally diagnosed as scrub typhus, except for patient 9 (suspected scrub typhus by mNGS with one specific reads of the pathogen) diagnosed as acute exacerbation of chronic obstructive pulmonary disease. For the five scrub typhus patients without typical eschar, mNGS gave all positive results (4–460 specific reads). For other methods, only Weil–Felix reaction of one patient detected the pathogen. In addition, the respiratory tract profile (IgM) detected various pathogens, but all were confirmed to be false positive.

**Conclusions:** mNGS performed better than conventional clinical methods to early diagnose scrub typhus. This approach can be routinely carried out for early and precise diagnosis in clinical infections, especially for those hard to be identified by traditional diagnostic methods.

## Introduction

Scrub typhus, also known as tsutsugamushi disease, is an acute febrile illness caused by *Orientia tsutsugamushi* (also referred to as *Rickettsia tsutsugamushi* or *Rickettsia orientalis*) (1). The pathogen is a kind of natural obligate intracellular parasite, which can be transmitted to humans by the bite of larval stage of certain kinds of trombiculid mites known as chiggers (2, 3). The disease was first described in Japan and estimated to have one million global cases per year, which seriously influenced the public health of Southeast Asia (3–5). Scrub typhus occurs seasonally, and most infections happen from July to August and from October to November in China (6). During 2006–2017, approximately 4,500 scrub typhus cases were diagnosed in Jiangxi Province, and more than 120,000 patients with scrub typhus were found in 30 provinces in China (7).

The clinical manifestations of this disease include fever, chills, skin rash, and eschar at the bite site. Patients with scrub typhus may also experience headache, myalgia, cough, generalized lymphadenopathy, nausea, vomiting, and abdominal pain (8). In severe cases, it may cause many complications of the lungs, brain, kidney, meninges, and heart, such as acute respiratory distress syndrome, acute kidney failure, encephalitis, gastrointestinal bleeding, meningitis, myocarditis, and pneumonia (9–11). The median mortality of scrub typhus is 6% in untreated patients and 1.45% in treated patients (12, 13). Its mortality can be much higher due to the complications in severe cases. For example, it can reach 14% in brain infections and 24% in patients with multiple organ dysfunction (13, 14). However, the early diagnosis of this infection is difficult, especially in patients without characteristic eschar. In fact, the presence of eschar ranges from 7 to 80% in different populations (15). Other rickettsial diseases may also cause eschar on body of patients, for example, Rocky Mountain spotted fever (16). The scrub typhus is usually misdiagnosed, due to its similar early symptoms to other acute febrile diseases. It needs a series of laborious techniques such as serology, biopsy, culture, and PCR to diagnose, however, all of which are insufficient in the diagnosis (17). For example, Weil–Felix reaction is fast and relatively easy to carry, but the results are always unfaithful (18). The cultural method is dangerous and is not available in most laboratories, which even takes an average of 4 weeks for identification (19). 16S rRNA and groEL gene real-time PCR performs high specificity, but the sensitivity has been reported to be from 45 to 82% (20).

Metagenomic next-generation sequencing (mNGS) is an unbiased approach and, theoretically, it detects all possible pathogens in one clinical sample. It is especially suitable for the diagnosis of rare, novel, and atypical etiologies of complicated infectious diseases (21). Compared to the routine diagnostic methods, mNGS always costs less time (<24 h) and can determine millions of base pairs untargeted sequences in a single experiment (22). As a rapid microbiological diagnostic method for infectious diseases, mNGS had been recently used in clinical practice due to its high efficiency, sensitivity, and cost-effectiveness (21). mNGS had also been introduced in the diagnosis of scrub typhus (23, 24).

In this study, blood and cerebrospinal fluid (CSF) samples of 10 patients with suspected scrub typhus were collected. mNGS and traditional test method were used for the early identification of the pathogen. We aim to evaluate the effectiveness of mNGS for the diagnosis of scrub typhus.

## Materials and Methods

### Patient Recruitment

A total of 10 patients with suspected scrub typhus were admitted to Ganzhou People's Hospital during May 2018 to March 2021. Most of the patients were farmers. Their symptoms included repeated fever (from 3 days to half a month), generalized lymphadenopathy, skin rash and eschar, abdominal pain, myalgia, headache, nausea, vomiting, and blurred vision. Most patients (9 out of 10) suffered repeated fever, and four patients had characteristic eschar. Blood tests were conducted immediately after hospitalization. Complete blood count including leukocytes, neutrophils, and lymphocytes counts were assessed. Liver and kidney function tests and coagulation tests of all patients were measured. The patients also received chest and abdomen computed tomography (CT) scanning and abdominal Color Doppler. Five patients received lumbar punctures, and Pandy's tests of all CSF samples were performed.

### Clinical Diagnostics

The body fluid samples of the suspected scrub typhus patients were collected, including blood, CSF, and pleural fluid. Widal reaction and Weil–Felix reaction were used to identify the presence of typhoid and rickettsia. Smear and blood cultures were also used for the detection of probable pathogens. The indirect immunofluorescence test (IIFT) of respiratory tract profile (IgM) (EUROIMMUN, China), including respiratory syncytial virus, adenovirus, influenza A virus, influenza B virus, parainfluenza virus, *Mycoplasma pneumoniae, Chlamydia pneumoniae*, anti-Coxsackievirus A type, anti-Coxsackievirus B type, Echo virus, and *Legionella pneumophila* were looked for. The suspected human cytomegalovirus, herpes simplex virus, and Epstein–Barr virus, which can cause similar clinical symptoms, were also analyzed using PCR in some patients.

### mNGS and qPCR Detection

A total of seven blood, five CSF samples, and one pleural fluid sample were collected for mNGS and qPCR analysis. Blood samples were collected and stored at 4°C. CSF and pleural fluid samples were drawn from patients and stored at −80°C. The samples were transmitted on dry ice for PACEseq mNGS detection (Hugobiotech, Beijing, China). QIAamp DNA Micro Kit (QIAGEN) was used to extract DNA according to its manual. DNA libraries were built using QIAseq Ultralow Input Library Kit (Illumina) based on the instructions of the manufacturer. We then analyzed the quality of all libraries using Qubit (Thermo Fisher) and Agilent 2,100 Bioanalyzer (Agilent Technologies). Libraries with high quality were finally sequenced on Nextseq 550 platform (Illumina). Reads <35 bp or with low quality (Q <30) were removed from the raw data. All human host DNA were filtered out by alignment to human reference database. The clean reads were finally aligned to Microbial Genome Databases (ftp://ftp.ncbi.nlm.nih.gov/genomes/) using software BWA. The probable pathogens and their mapped reads numbers were reported. qPCR was also performed using *O. tsutsugamushi* real-time PCR Kit (Liferiver) based on its manual (25) on Applied Biosystems 7,500 Real-Time PCR System (ABI). The detection results between qPCR and mNGS were compared using the Chi-square test. The value of mNGS in early diagnosing scrub typhus was evaluated.

## Results

### Patient Characteristics

Demographic features and baseline data of the patients are shown in Supplementary Table 1. Of the 10 patients, 6 were women and 4 were men. The mean age was 64.8 years (44–82 years). Seven out of 10 patients were farmers. For the other three patients, one was only known to live in the countryside, the other two had no relevant information. Half of the patients experienced underlying diseases, including diabetes, atrial fibrillation, thyroid nodules, chronic bronchitis, and coronary heart disease. Fever was the main symptom, which ranged from 3 to 15 days in 9 patients. The concomitant symptoms included generalized lymphadenopathy, abdominal pain, myalgia, headache, nausea, vomiting, and blurred vision. Iconography studies showed hepatosplenomegaly in six patients and pulmonary infections in eight patients. Dysfunctions of multiple organs were found in these patients, including lungs (three respiratory failures), heart (four heart failures), liver (eight liver damages), and kidneys (one kidney damage). Pandy's tests of CSF found that two out of seven patients presented positive, indicating infections in meninges. The main symptoms of the patients were similar to those in acute febrile diseases. Only four patients (40%) had ulcer or eschar on body, which helped their diagnosis as suspected scrub typhus. The clinical characteristics of patients are shown in Table 1.

**Table 1:** The clinical characteristics of the patients of this study.

**Patient**	**Main symptoms**	**Skin rash and eschar**	**Hepatosplenomegaly**	**Swollen lymph nodes**
Patient 1	Repeated fever (~10 days)	No	Yes	NA
Patient 2	Fever, abdominal pain, and palpitations (3 days)	No	No	Neck
Patient 3	Repeated fever (5 days), and myalgia (~10 days)	Yes	Yes	Neck
Patient 4	Repeated fever (7 days)	Yes	No	Right groin
Patient 5	Fever (10 days)	Yes	Fatty liver	NA
Patient 6	Fever (15 days)	Yes	Splenomegaly	Double armpit
Patient 7	Fever (7 days), dizziness, nausea, and vomiting	No	Splenomegaly	Double armpit
Patient 8	Fever (~10 days), and abdominal pain (one month)	No	Splenomegaly	NA
Patient 9	limbs fatigue (7 days), and blurred vision	No	Liver cyst	Lungs, retroperitoneum, and mesenteric root
Patient 10	Fever, and headache (9 days)	No	Yes	NA

### Conventional Detection Results

Widal reaction and Weil–Felix reaction were employed for seven patients. Only one was detected to be scrub typhus with a four-fold rise in titers by Weil–Felix reaction. The sensitivity of Weil–Felix reaction was 11.1%. To detect other probable pathogens, blood culture and smear of nine patients were performed. The results were all negative. The respiratory tract profile (IgM) was used in five patients to detect probable respiratory tract infections. Only one patient (patient 3) was negative, the other four provided positive results. The detected pathogens included the respiratory syncytial virus and parainfluenza virus in patient 1, *Legionella* in patient 4, *Mycoplasma pneumoniae* in patient 6, and influenza A virus and parainfluenza virus in patient 9. However, the following nucleic acid tests of throat swab indicated that these results were all false positive. In addition, PCR analysis of human cytomegalovirus, herpes simplex virus, and Epstein–Barr virus in three patients showed all negative. The detailed results of conventional detection are shown in Table 2.

**Table 2:** The results of diagnosis by different methods.

**Patient**	**Widal reaction**	**Weil Felix reaction**	**Blood cultures and smear**	**Respiratory pathogen spectrum**
Patient 1	Negative	Negative	Negative	Respiratory syncytial virus, and parainfluenza virus
Patient 2	Negative	Negative	Negative	NA
Patient 3	Negative	Negative	Negative	Negative
Patient 4	Negative	Negative	Negative	*Legionella*
Patient 5	NA	NA	Negative	NA
Patient 6	Negative	Negative	Negative	*Mycoplasma pneumoniae*
Patient 7	NA	NA	Negative	NA
Patient 8	Negative	Negative	Negative	NA
Patient 9	NA	NA	NA	Influenza A virus
Patient 10	Negative	Positive	Negative	NA

### mNGS and qPCR Results

**Figure 1:**
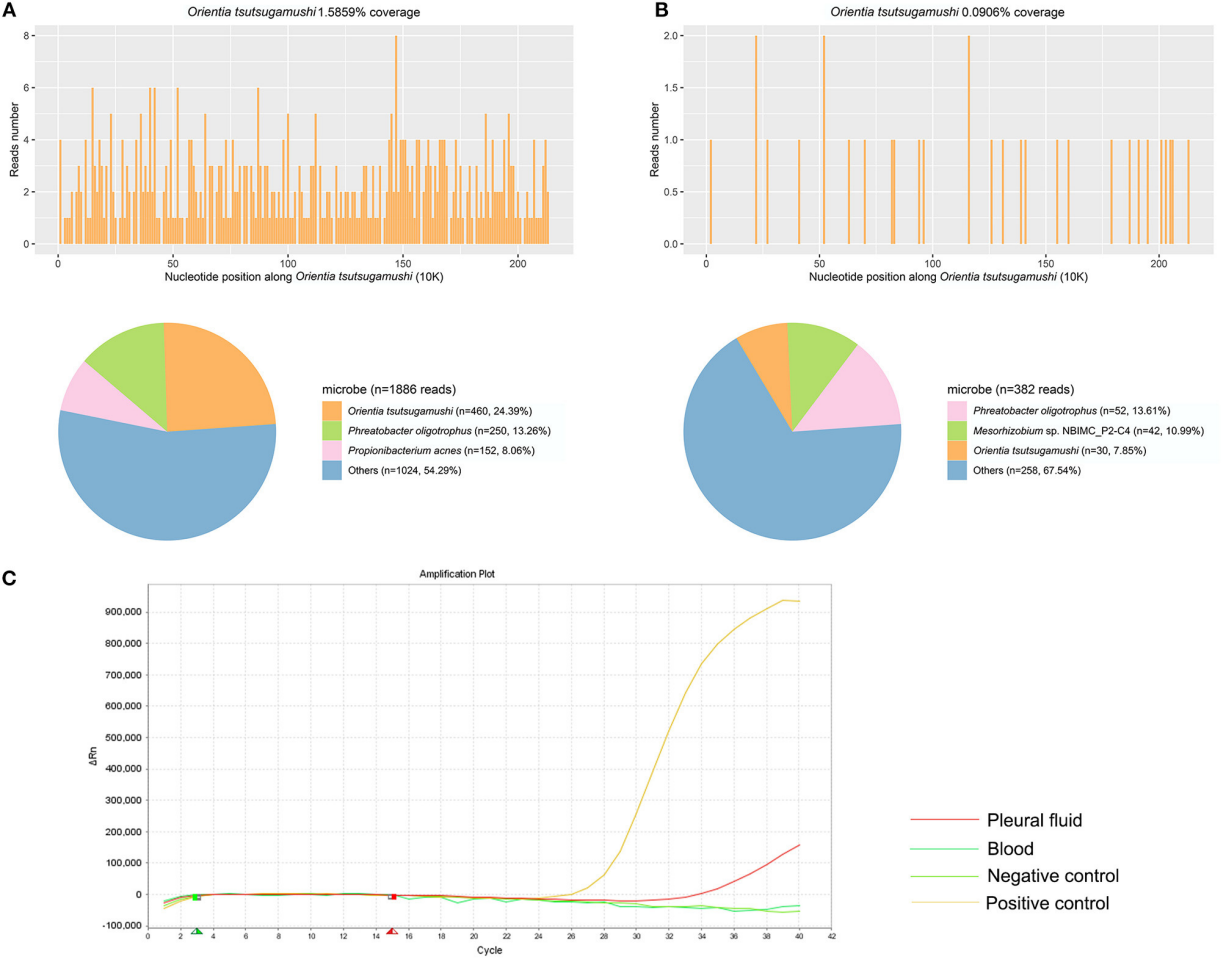
The results of metagenomic next-generation sequencing (mNGS) and qPCR in patient 8 using pleural fluid and blood. **(A)** A total of 460 unique reads of *Orientia tsutsugamushi* were detected by mNGS using pleural fluid. **(B)** A total of 30 unique reads of *O. tsutsugamushi* were detected by mNGS using blood. **(C)** qPCR of *O. tsutsugamushi* showed positive in pleural fluid but negative in blood.

Thirteen samples of the 10 patients were used for mNGS, including blood, CSF, and pleural fluid. The unique reads of *O. tsutsugamushi* were detected in all samples (Supplementary material). Eight patients were diagnosed as scrub typhus by mNGS. The number of unique sequences of *O. tsutsugamushi* ranged from 4 to 460. Two patients were diagnosed as suspected scrub typhus due to the small number of *O. tsutsugamushi* reads (one and two reads, respectively) with medium confidence. The sensitivity of mNGS was 100%. qPCR was subsequently used to confirm the results of mNGS. Only one sample (the pleural fluid of patient 8) was detected to be positive (Figure 1). The sensitivity of qPCR was 11.1%. Interestingly, this sample was also detected with the most pathogen reads (460 reads) by mNGS. The mNGS and qPCR results are shown in Table 3 and Supplementary Figures.

**Table 3:** The types of samples and the results of mNGS and qPCR.

**Patient**	**Type of sample**	**Number of reads by mNGS**	**qPCR results**
Patient 1	CSF	10	Negative
Patient 2	CSF	10	Negative
	Blood	4	Negative
Patient 3	Blood	4	Negative
Patient 4	Blood	32	Negative
Patient 5	Blood	6	Negative
Patient 6	Blood	2	Negative
Patient 7	CSF	24	Negative
	Blood	50	Negative
Patient 8	Pleural fluid	460	Positive
	Blood	30	Negative
Patient 9	CSF	1	Negative
Patient 10	CSF	66	Negative

*CSF, cerebrospinal fluid; mNGS, metagenomic next-generation sequencing*.

mNGS results of different sample types were also compared in this study. No significant difference of specific reads of the pathogen was found in mNGS results between blood samples (7) and CSF samples (5) (*p* > 0.05). Of patient 2 and patient 7, the blood and CSF samples were collected at the same time for mNGS. The number of pathogen reads detected in patient 2 from blood and CSF samples were 4 and 10, respectively. And for patient 7, there were 50 and 24 specific reads of the pathogen detected in blood and CSF samples, respectively. It seemed that there was no significant difference between the samples of blood and CSF for mNGS of this pathogen. The efficiencies of mNGS using blood and pleural fluid samples were also compared in patient 8. A total of 460 pathogen reads were detected using pleural fluid sample, while only 30 were found using blood sample.

Of the 10 patients, 9 were finally diagnosed as scrub typhus. While patient 9 (only one unique read of *O. tsutsugamushi*) was identified to be acute exacerbation of chronic obstructive pulmonary disease with coronary heart disease, chronic heart failure (NYHA level 3), silicosis, and old tuberculosis. Patient 6 was also diagnosed as suspected scrub typhus by mNGS with media confidence due to its low detection of pathogen reads, but there was eschar on his body. He was also confirmed to be scrub typhus by final clinical evidences. In addition, except one patient who did not suffer scrub typhus, five scrub typhus patients without typical eschar were all confirmed by mNGS, which was performed with high sensitivity and specificity in the early diagnosis of scrub typhus.

Upon admission, the patients with typical eschar were treated with doxycycline directly. While for the patients without eschar, bacterial infections were first considered. Cephalosporins were used during the treatments, but the effects were limited. When mNGS results came out, their treatments were adjusted immediately to doxycycline. The symptoms of the patients improved gradually, and their temperatures returned to normal in 1–3 days. All the patients fully recovered and most of them were discharged in 7–10 days.

## Discussion

In this study, we enrolled 10 patients with suspected scrub typhus, nine of whom were finally diagnosed as this disease. Concomitant pneumonia, dysfunctions of multiple organs, and encephalitis were found in these patients, of which pneumonia was the most common one. Scrub typhus is widespread in China and has become a burden on public health and economy. The deteriorated infection can also cause multiple-organ failure and even death. The mortality of scrub typhus in severe cases or due to improper treatment or misdiagnosis can reach 30–70% (12). Therefore, a better method of timely distinction is always needed.

Weil–Felix is the simplest and fastest serological test, which is often used to detect all kinds of rickettsial diseases. However, the result is notoriously unreliable due to its low positive rate in the early diagnosis (26). In this study, Weil–Felix test was used for seven patients and only one was found positive. The sensitivity of Weil–Felix reaction was very low. Culture was always applied to identify pathogens of different infections, but it had limitations for some microorganisms. The culture of *O. tsutsugamushi* was dangerous and not conducted in this study. The respiratory tract profile (IgM) always gave false-positive results in this study. Considering the untypical symptoms of scrub typhus, this disease was hard to be early diagnosed in older patients and those with no obvious eschar on body, especially when the patients had underlying diseases. The scrub typhus could be always misdiagnosed to other acute febrile diseases, such as malaria, leptospirosis, typhoid, and dengue fever (27, 28). Notably, in our study, 6 out of 10 patients were without eschar and could not be diagnosed by routine clinical methods. In addition, a diagnosis of simple pulmonary infection might have happened due to the presence of *Legionella* and *Mycoplasma pneumoniae* by the respiratory tract profile (IgM) combined with iconography evidences in two patients. Fortunately, mNGS provided faithful results and helped the diagnosis of these patients. The patients received timely specific treatment and were finally discharged with a good prognosis.

qPCR was previously considered as a good tool for the detection of scrub typhus and was usually used in the diagnosis of this disease (20). However, in this study, qPCR performed with a quite low detection rate, with only the pleural fluid sample of patient 8 showing positive with the cycle threshold (CT) at 34.2 cycles. Nevertheless, her blood sample displayed a negative qPCR result. Interestingly, we found that the pleural fluid sample with a positive qPCR result also had the largest number of pathogen reads by mNGS among all the 13 samples. All other samples that were positive with mNGS test were negative for the qPCR test, indicating a better level of detection of mNGS than qPCR methods. The positive detection rate of Weil–Felix reaction in our study was also quite low. One probable reason for the significantly lower sensitivity of qPCR and Weil–Felix reaction than mNGS (*p* < 0.01) was that the early detection was performed within 2 weeks (3–15 days) of the onset of symptoms. The patients only suffered mild symptoms of scrub typhus, and the content of the pathogen in the samples might be not enough to get positive results. For example, Weil–Felix reaction only detected a titer of 1:20 in patient 4.

Compared with the conventional methods, mNGS showed a higher sensitivity. In this study, all the samples of the patients were positive, with the number of *O. tsutsugamushi* reads ranging from 1 to 460. The results of eight patients showed high confidence level, which indicated scrub typhus. The patient with two unique reads of the pathogen was also confirmed as scrub typhus due to the eschar on body and final clinical evidences. Only the patient with one specific read was identified to be other disease. mNGS was successfully applied to the diagnosis of clinical cases, including immunocompromised, meningitis, pneumonia, and sepsis (29–31). It demonstrated a better ability in the diagnosis of infections by rare, novel, and complex pathogens (21).

Different types of samples including CSF, blood, bronchoalveolar lavage fluid, tissue, pleural fluid, throat swab, and other body fluid were used for mNGS, among which, CSF and blood samples were more common. In this study, we compared the performance of blood, CSF, and pleural fluid samples for mNGS in the same patient. In patient 2, the detected number of reads of *O. tsutsugamushi* using CSF sample was higher than blood sample. While in patient 7, the result was opposite. We did not see any difference of mNGS by CSF and blood samples. However, in patient 8, pleural fluid sample performed better than blood sample. More samples of different types from the same patients are needed for further analysis.

There are some limitations. The sample size of this study is small. The disease is not common. Only 4,500 scrub typhus cases were diagnosed in Jiangxi Province during 2006–2017. Its symptoms are atypical, and most infected patients are farmers, who prefer to receive treatments in local hospitals. This may cause a large amount of misdiagnoses and miss diagnoses. In addition, due to the relatively high price of mNGS, most patients did not receive mNGS detection. Moreover, although conventional diagnostic methods, including serology and qPCR were detected, the results were limited. Fortunately, when considering the presence of the typical symptom (eschar) and positive results from traditional methods of this disease, six (four with eschar, one only with positive Weil–Felix result, and one only with positive qPCR result) of the nine patients could be diagnosed with scrub typhus, which further confirmed the mNGS detection.

## Conclusions

The precise and timely treatment is important for the survival of patients with scrub typhus. But the early diagnosis of this infection is difficult, especially for patients without eschar. Misdiagnosis and miss diagnosis of the disease often occur due to its similar symptoms with other acute febrile illnesses. In this study, mNGS was introduced for the early diagnosis, which performed faster and more sensitive than the other clinical diagnosis methods. Thus, mNGS can be a promising technology for early and precise diagnosis in clinical infections, for scrub typhus, and for those hard to be identified by traditional diagnosis methods.

## Data Availability Statement

The datasets presented in this study can be found in online repositories. The names of the repository/repositories and accession number(s) can be found in the article/Supplementary Material.

## Ethics Statement

The studies involving human participants were reviewed and approved by the Ethical Review Committee of Ganzhou People's Hospital. The patients/participants provided their written informed consent to participate in this study.

## Author Contributions

XL, YZ, and ZLu designed and drafted the manuscript. XL and JZ involved in the clinical care and management of the patients. ZLo and HX analyzed the mNGS data. All authors approved the final manuscript as submitted and agreed to be accountable for all aspects of the work.

## Conflict of Interest

YZ, ZL, and HX are employed by Hugobiotech Co., Ltd. The remaining authors declare that the research was conducted in the absence of any commercial or financial relationships that could be construed as a potential conflict of interest.

## Publisher's Note

All claims expressed in this article are solely those of the authors and do not necessarily represent those of their affiliated organizations, or those of the publisher, the editors and the reviewers. Any product that may be evaluated in this article, or claim that may be made by its manufacturer, is not guaranteed or endorsed by the publisher.

## Supplementary Material

Supplementary Figures 1–11 The mNGS results of patients 1–10 are shown in Supplementary Figures 1–10. The qPCR results were shown in Supplementary Figure 11.

Supplementary Table 1 Demographic features and baseline data of the patients in this study are shown in Supplementary Table 1. The detailed information of the detected reads of *Orientia tsutsugamushi* are shown in Supplementary Tables.
