# Supplementary material for: The Early Diagnosis of Scrub Typhus by Metagenomic Next-Generation Sequencing
Source: Front Public Health. 2021 Nov 11;9:755228. doi: 10.3389/fpubh.2021.755228 (PMC8632043; doi:10.3389/fpubh.2021.755228)

Figure S1. The detected result of Patient 1 by mNGS using CSF.


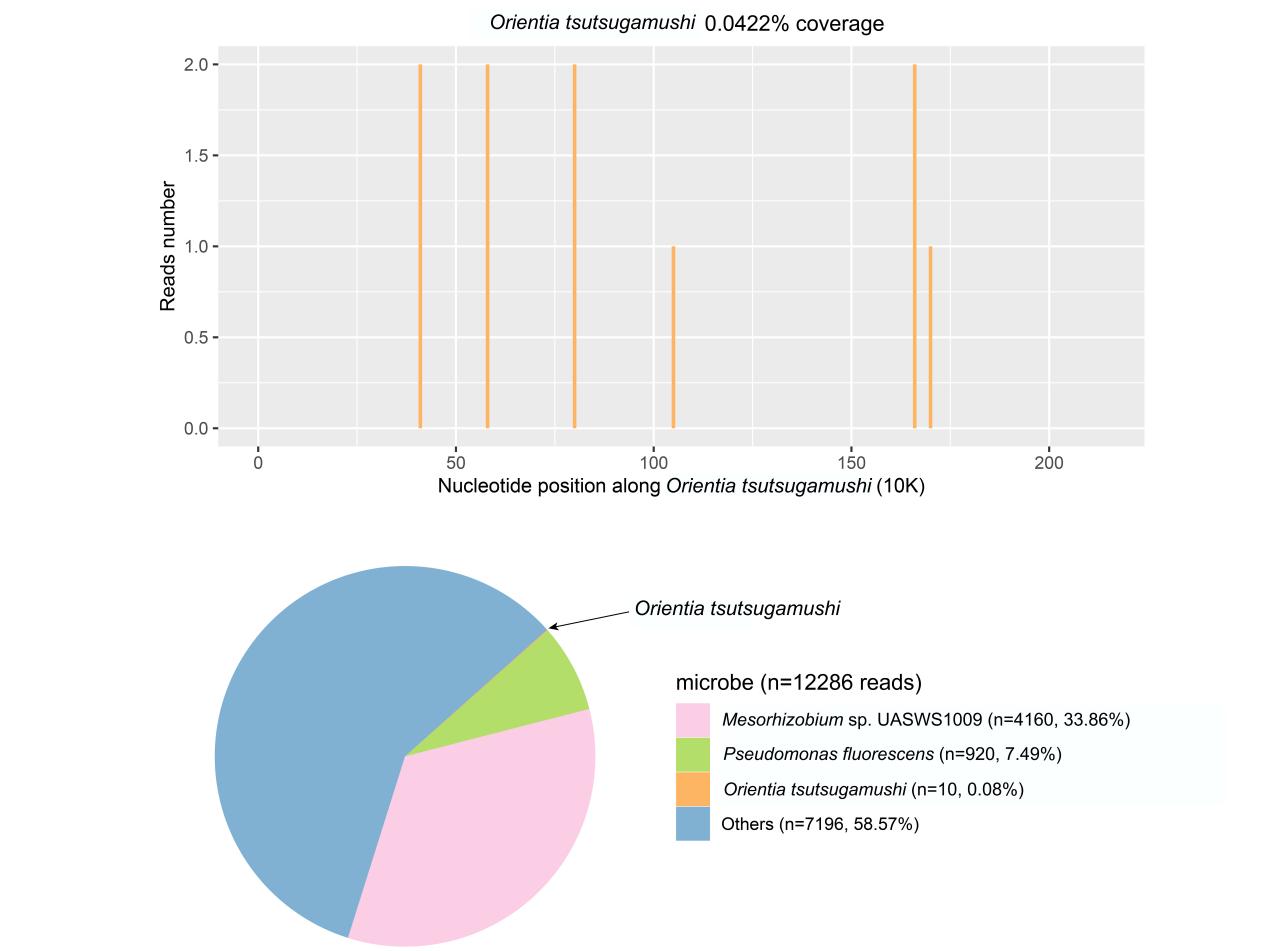


Figure S2. The detected result of Patient 2 by mNGS using CSF and blood.


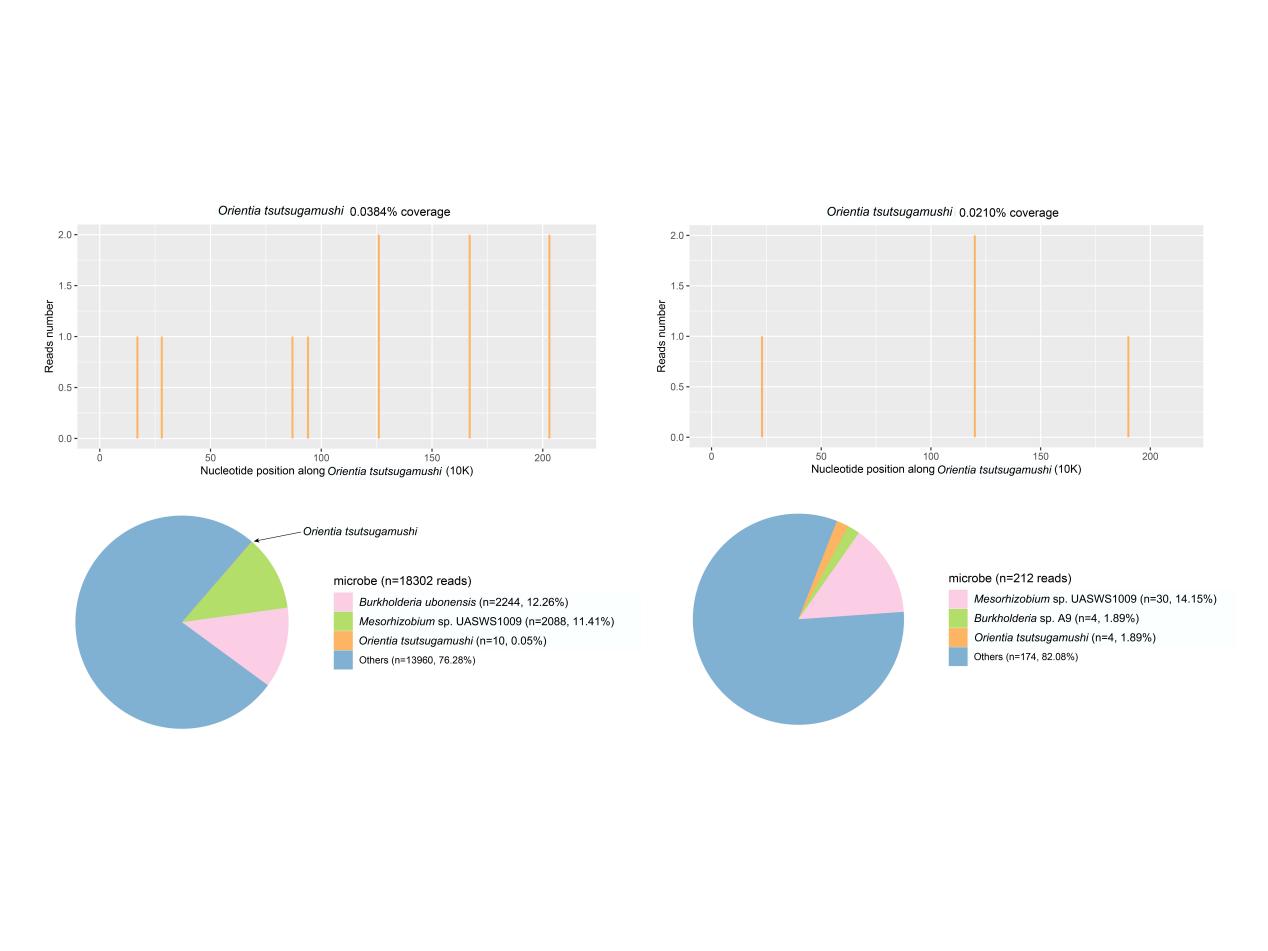


Figure S3. The detected result of Patient 3 by mNGS using blood.


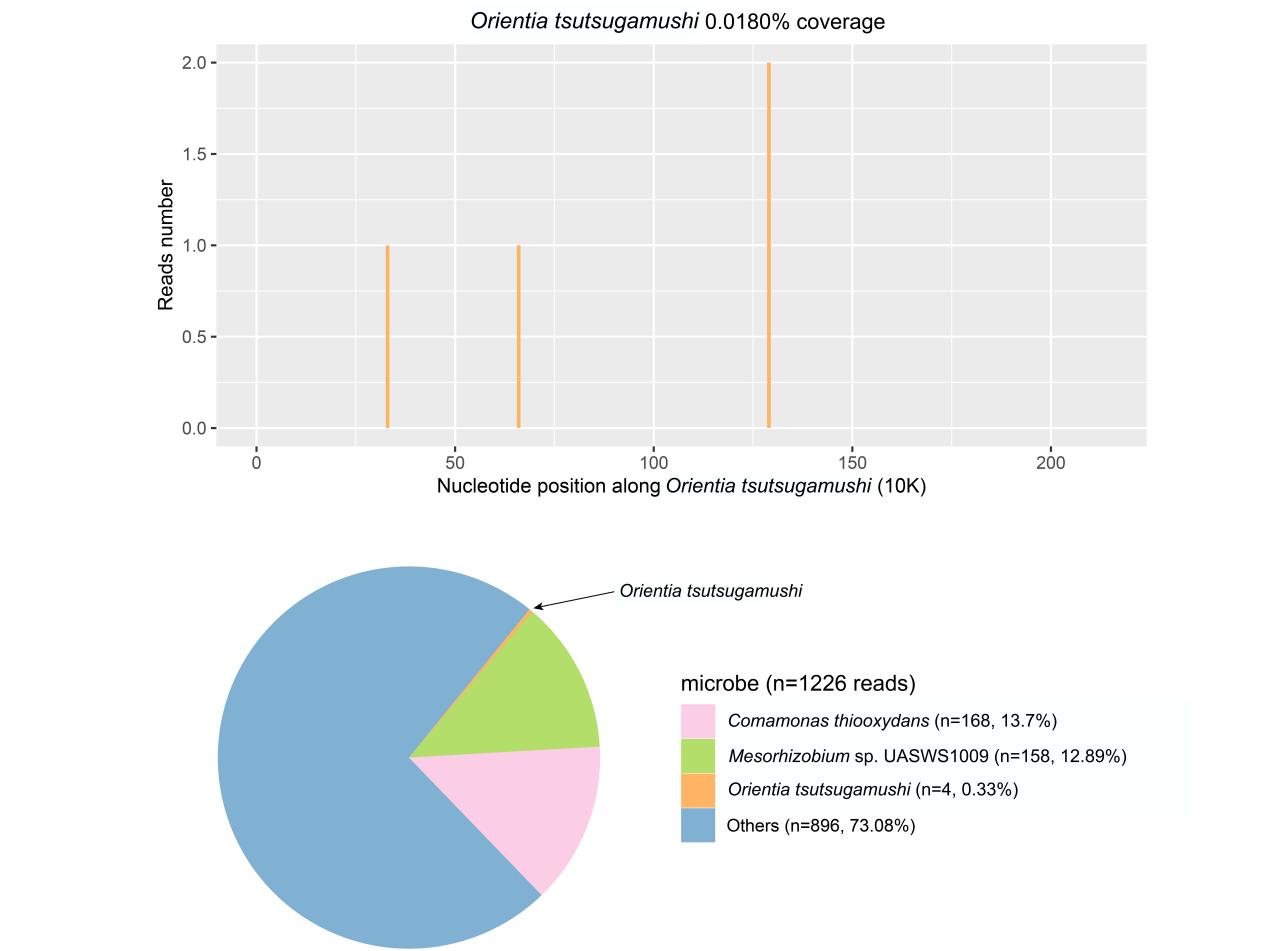


Figure S4. The detected result of Patient 4 by mNGS using blood.


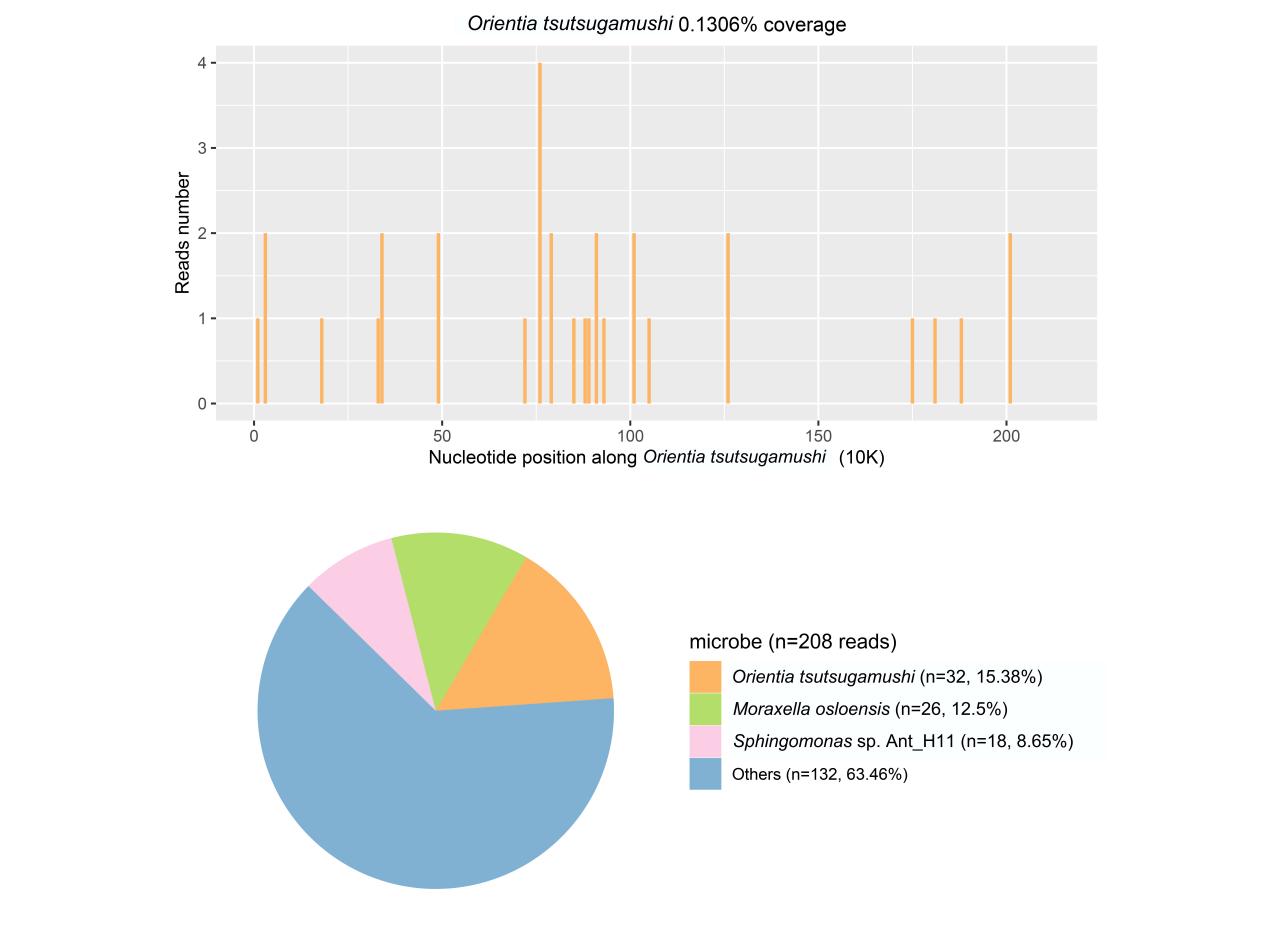


Figure S5. The detected result of Patient 5 by mNGS using blood.


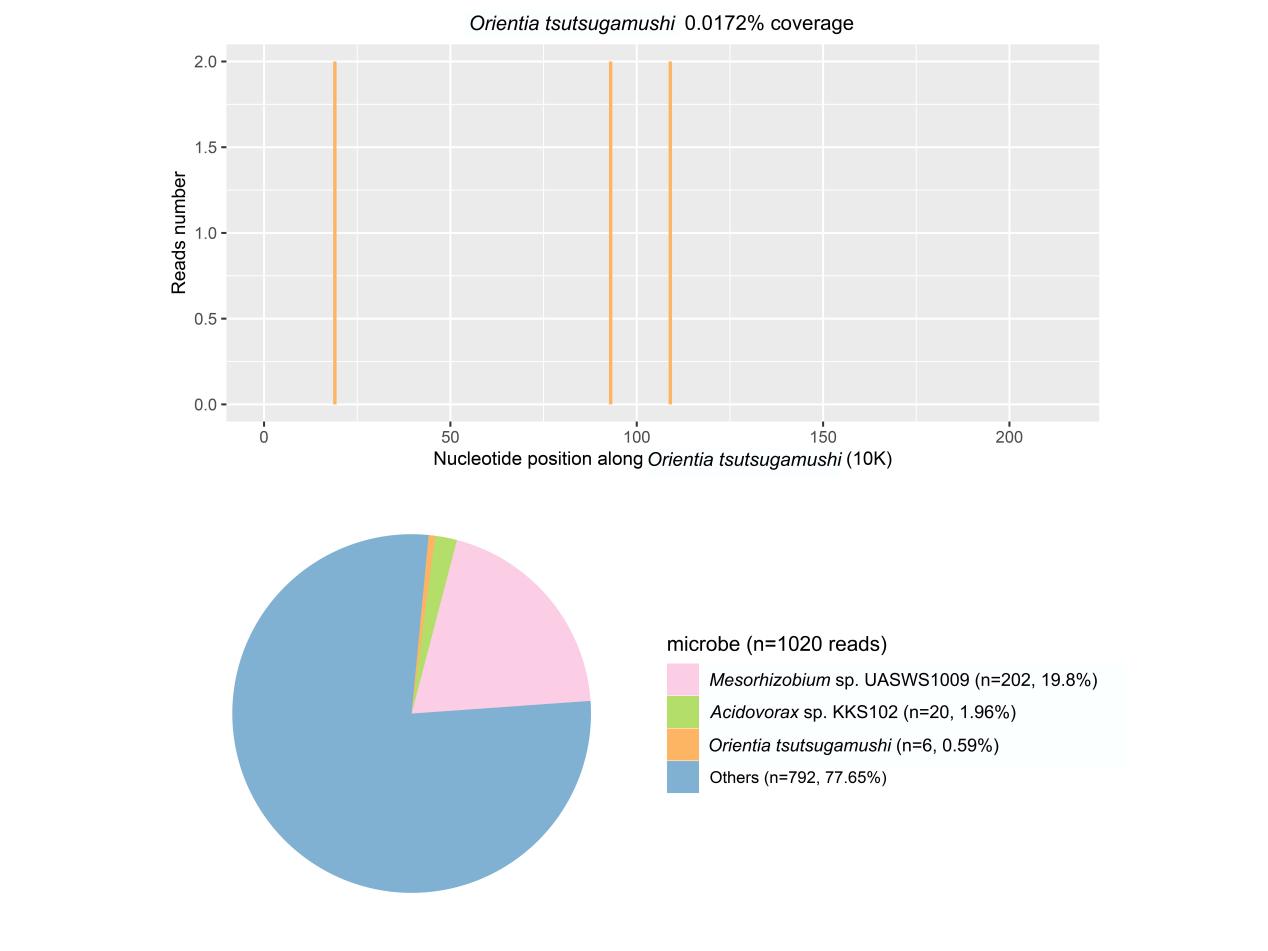


Figure S6. The detected result of Patient 6 by mNGS using blood.


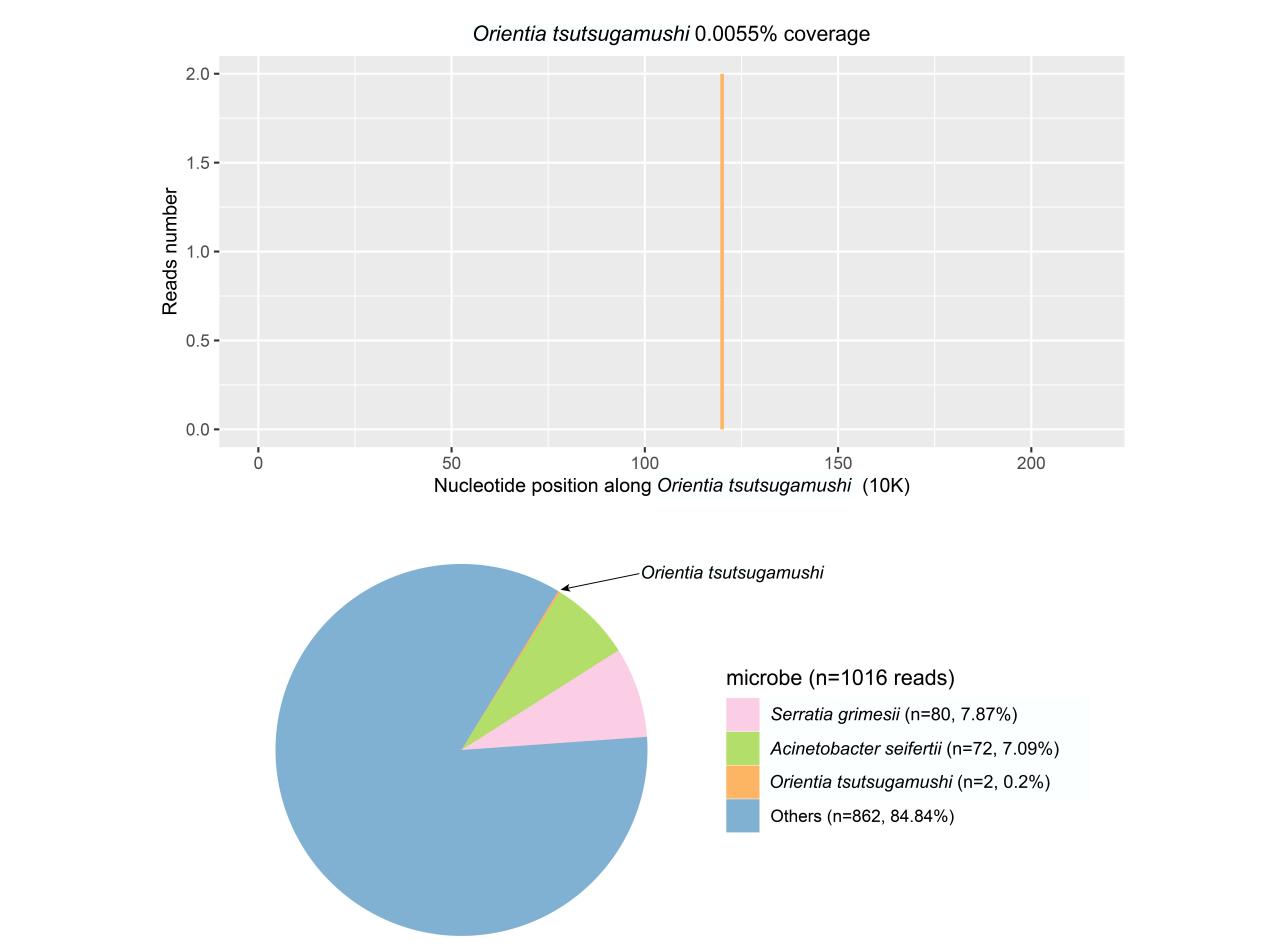


Figure S7. The detected result of Patient 7 by mNGS using CSF and blood.


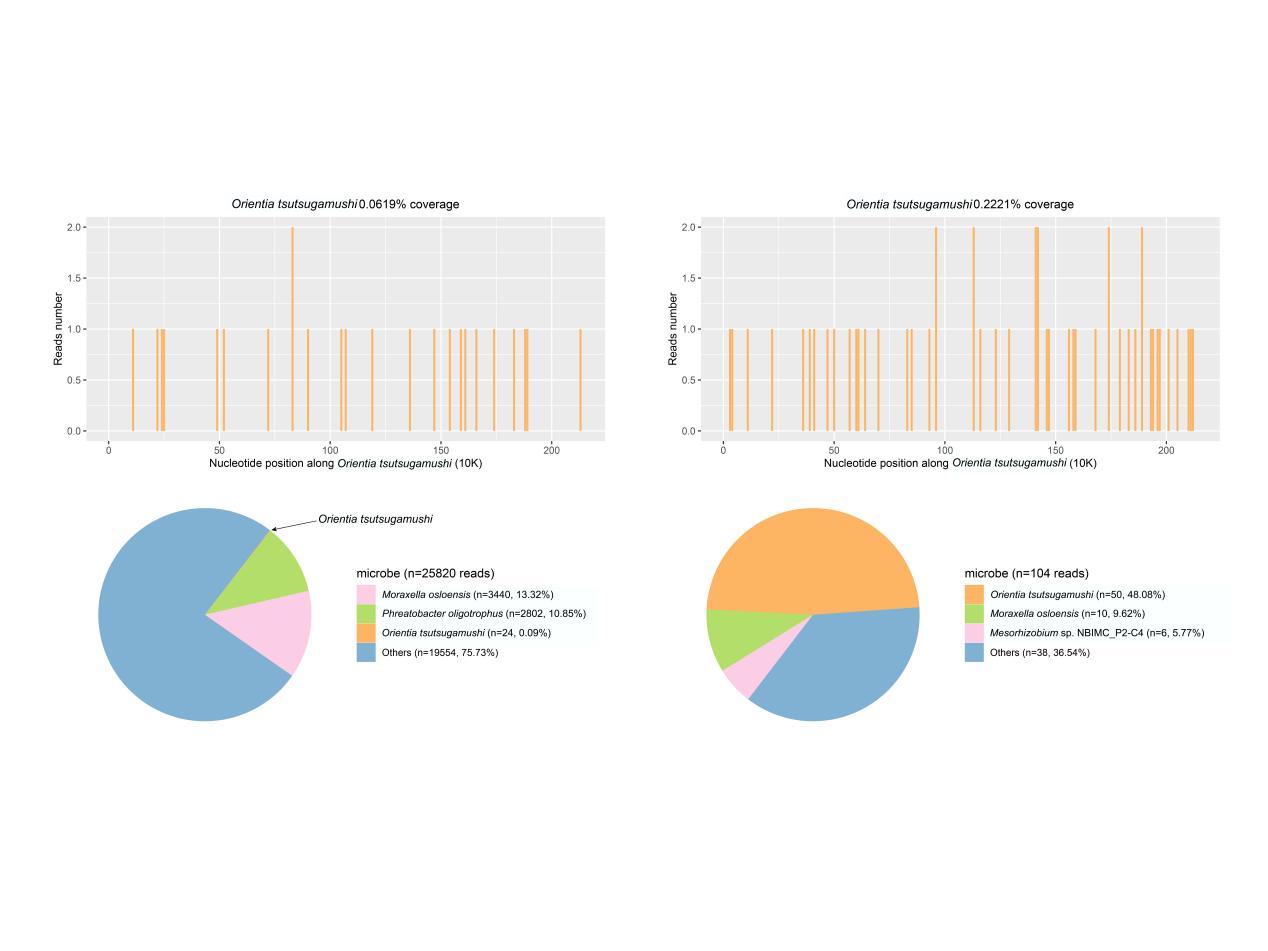


Figure S8. The detected result of Patient 8 by mNGS using pleural fluid and blood.





Figure S9. The detected result of Patient 9 by mNGS using CSF.


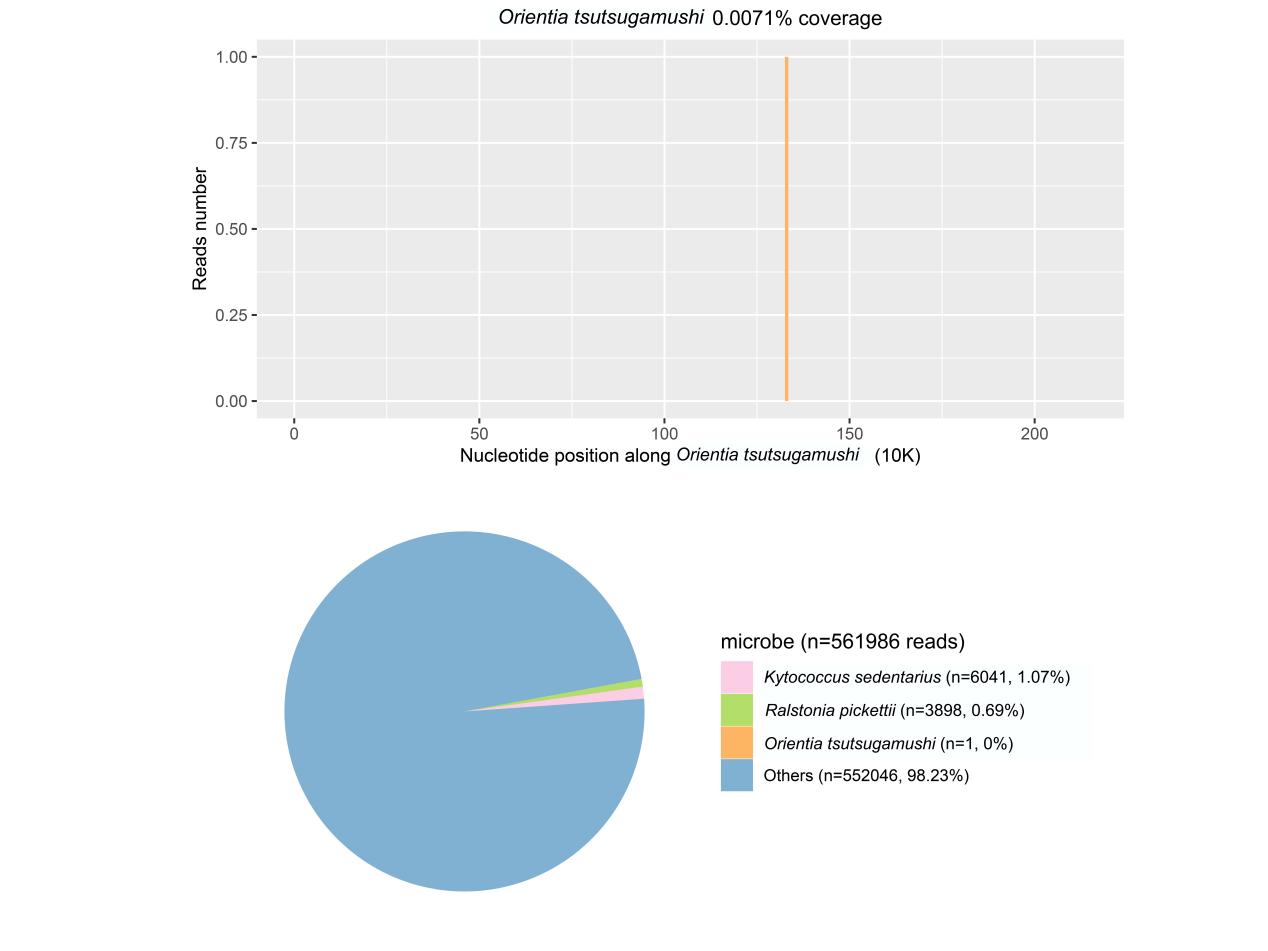


Figure S10. The detected result of Patient 10 by mNGS using CSF.





Figure S11. The detected results of qPCR.


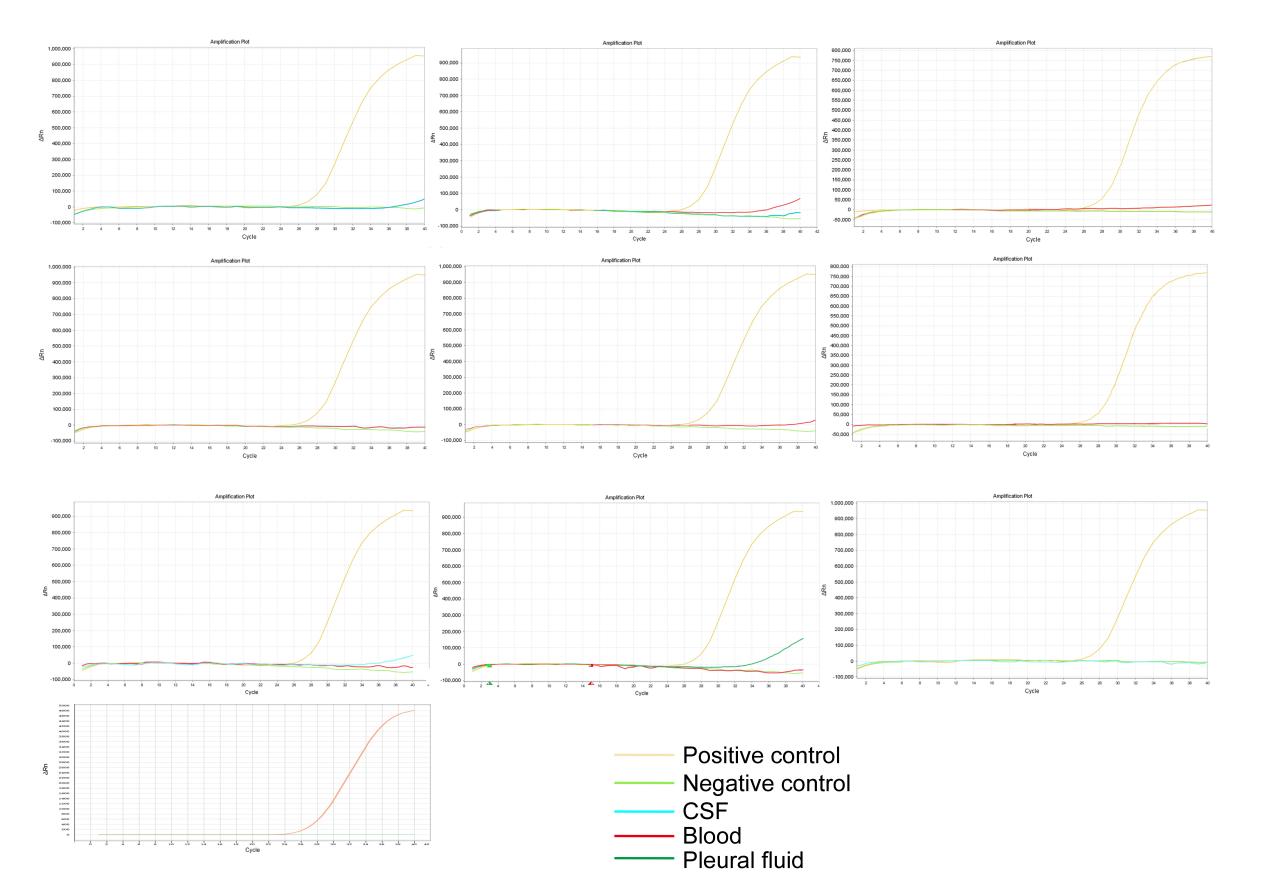

Supplement: Supplementary Figures 1–11 — The mNGS results of patients 1–10 are shown in Supplementary Figures 1–10. The qPCR results were shown in Supplementary Figure 11. [file Data_Sheet_1.docx]
